# Supplementary material for: In-situ and wavelength-dependent photocatalytic strain evolution of a single Au nanoparticle on a TiO2 film
Source: Nat Commun. 2024 Jun 27;15:5416. doi: 10.1038/s41467-024-49862-1 (PMC11211407; doi:10.1038/s41467-024-49862-1)
Supplement: Supplementary file 1 — Supplementary Information [file 41467_2024_49862_MOESM1_ESM.pdf]

Supplementary Information

In-situ and wavelength-dependent photocatalytic strain evolution of a single Au nanoparticle on a TiO_2 film

*Sunghyun Park[1], Sukyoung Kim[2,3], Jae Whan Park[4], Seunghee Kim[2,3], Wonsuk Cha[5], Joonseok Lee[2,3,6]**

[1]Department of HY-KIST Bio-Convergence, Hanyang University, Seoul 04763, Republic of Korea

[2]Department of Chemistry, Hanyang University, Seoul 04763, Republic of Korea

[3]Research Institute for Natural Sciences, Hanyang University, Seoul, 04763, Republic of Korea

[4]Center for Artificial Low Dimensional Electronic Systems, Institute for Basic Science, Pohang 37673, Republic of Korea

[5]X-ray Science Division, Advanced Photon Source, Argonne National Laboratory, Argonne, IL 60439, USA

[6]Research Institute for Convergence of Basic Sciences, Hanyang University, Seoul 04763, Republic of Korea

***Corresponding authors**

E-mail address: joonseoklee@hanyang.ac.kr

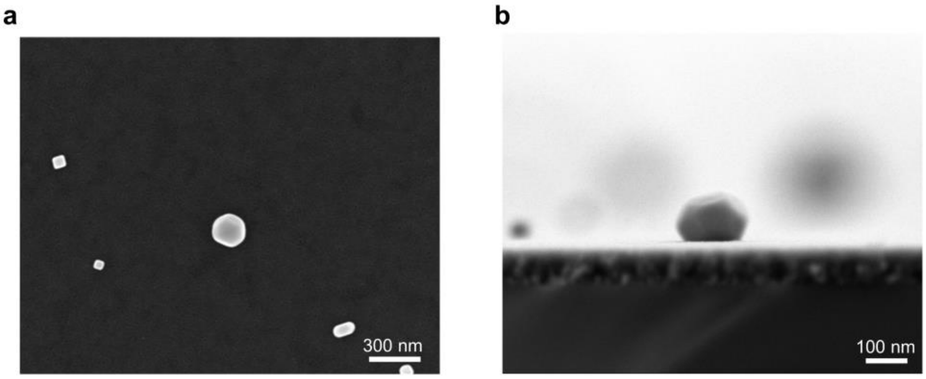

Supplementary Fig. 1. SEM images of the Au/TiO_2 heterostructure. The SEM images of **a** top view and **b** cross-view show the AuNPs on the TiO_2 substrate. The focused X-ray beam was illuminated to an isolated AuNP in BCDI measurements.

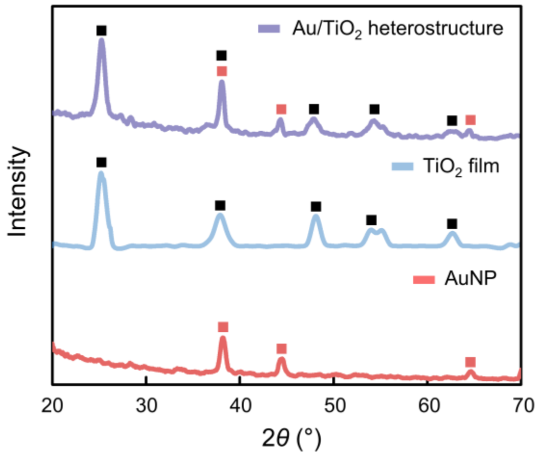

Supplementary Fig. 2. Structure characterization. XRD patterns of Au/TiO_2 heterostructure, TiO_2 (JCPDS No. 21-1272), and AuNP (JCPDS No. 04-0784) with peaks labeled based on the standard pattern of the pure phase of Au and TiO_2. Colors in purple, blue, and red represent Au/TiO_2 heterostructure, TiO_2 film, and AuNP respectively. Source data are provided as a Source Data file.

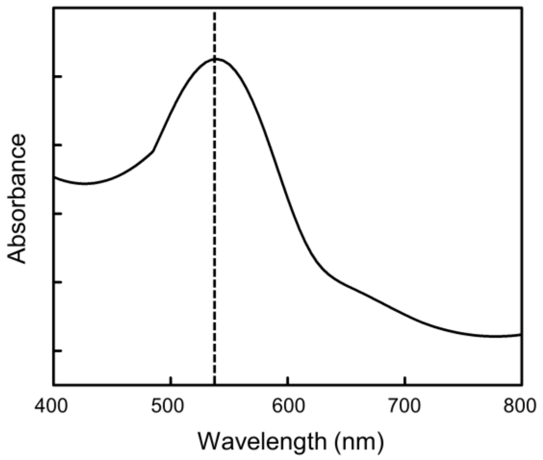

Supplementary Fig. 3. UV-vis spectra of AuNP. The observed peak of 532 nm indicates the maximum absorbance wavelength of AuNP. Source data are provided as a Source Data file.

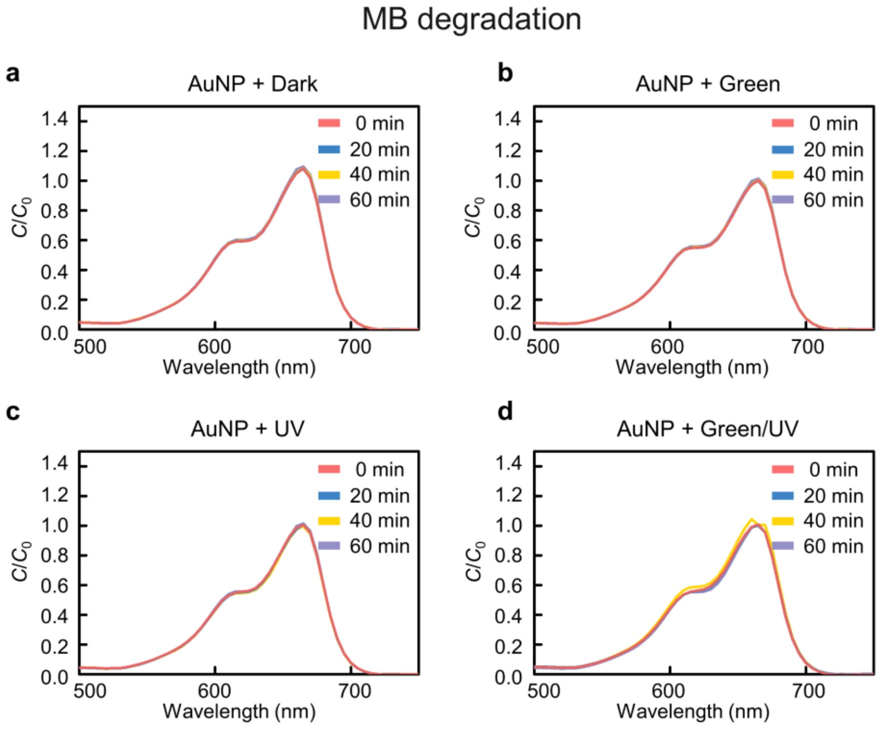

Supplementary Fig. 4. **MB degradation test of AuNP.** Photocatalytic degradation of MB on AuNP under **a** dark, **b** green, **c** UV, and **d** green/UV irradiation for 60 min. Colors in red, blue, yellow, and purple represent 0 min, 20 min, 40 min, and 60 min irradiation, respectively. Source data are provided as a Source Data file.

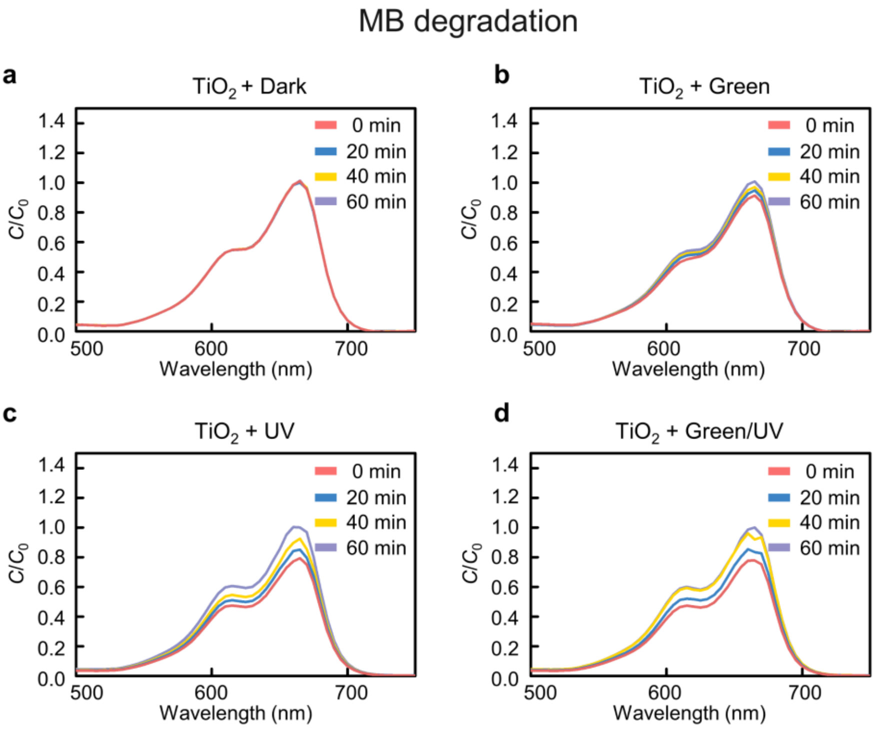

Supplementary Fig. 5. **MB degradation test of TiO_2.** Photocatalytic degradation of MB on TiO_2 under **a** dark, **b** green, **c** UV, and **d** green/UV irradiation for 60 min. Colors in red, blue, yellow, and purple represent 0 min, 20 min, 40 min, and 60 min irradiation, respectively. Source data are provided as a Source Data file.

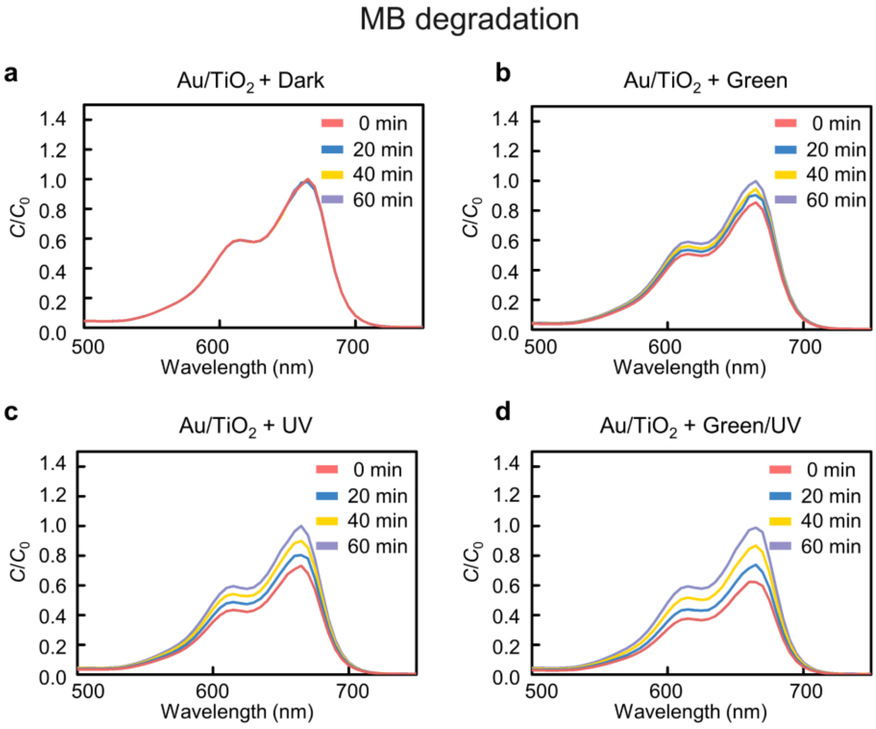

Supplementary Fig. 6. **MB degradation test of Au/TiO_2.** Photocatalytic degradation of MB on Au/TiO_2 under **a** dark, **b** green, **c** UV, and **d** green/UV irradiation for 60 min. Colors in red, blue, yellow, and purple represent 0 min, 20 min, 40 min, and 60 min irradiation, respectively. Source data are provided as a Source Data file.

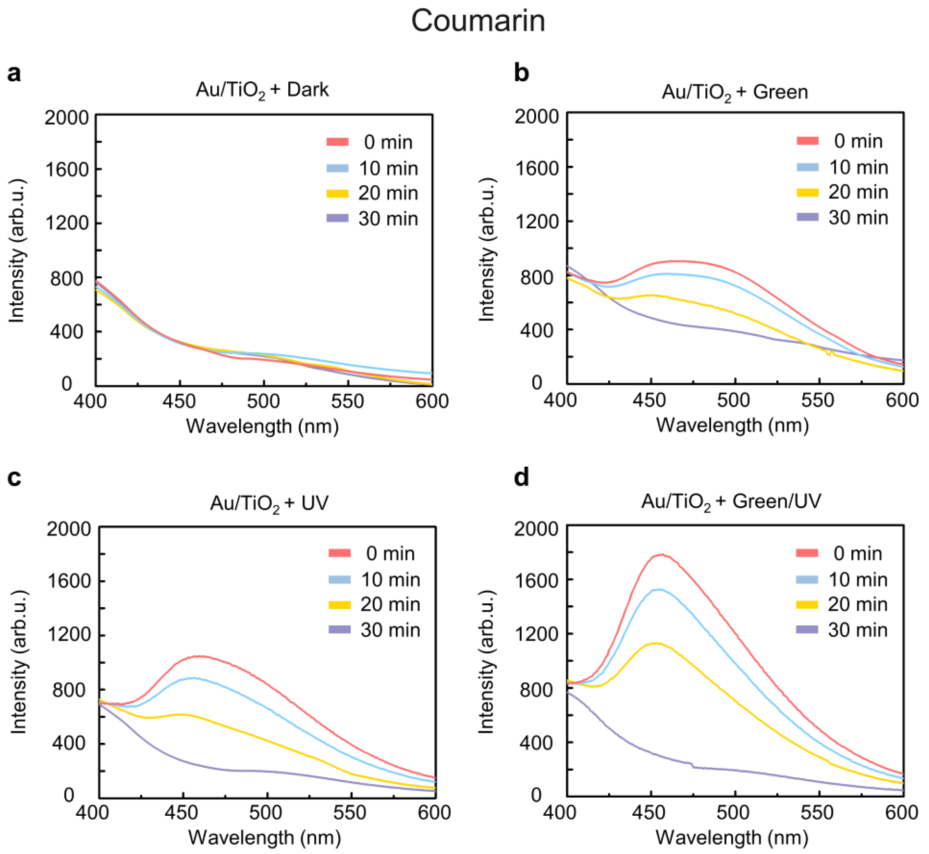

Supplementary Fig. 7. Fluorescence intensity of 7-OH-coumarin. Time-dependent fluorescence intensity of 7-OH-coumarin for detecting the •OH generation of the Au/TiO_2 heterostructure irradiated by **a** dark, **b** green, **c** UV, and **d** green/UV. arb.u., arbitrary units. Colors in red, blue, yellow, and purple represent 0 min, 20 min, 40 min, and 60 min irradiation, respectively. Source data are provided as a Source Data file.

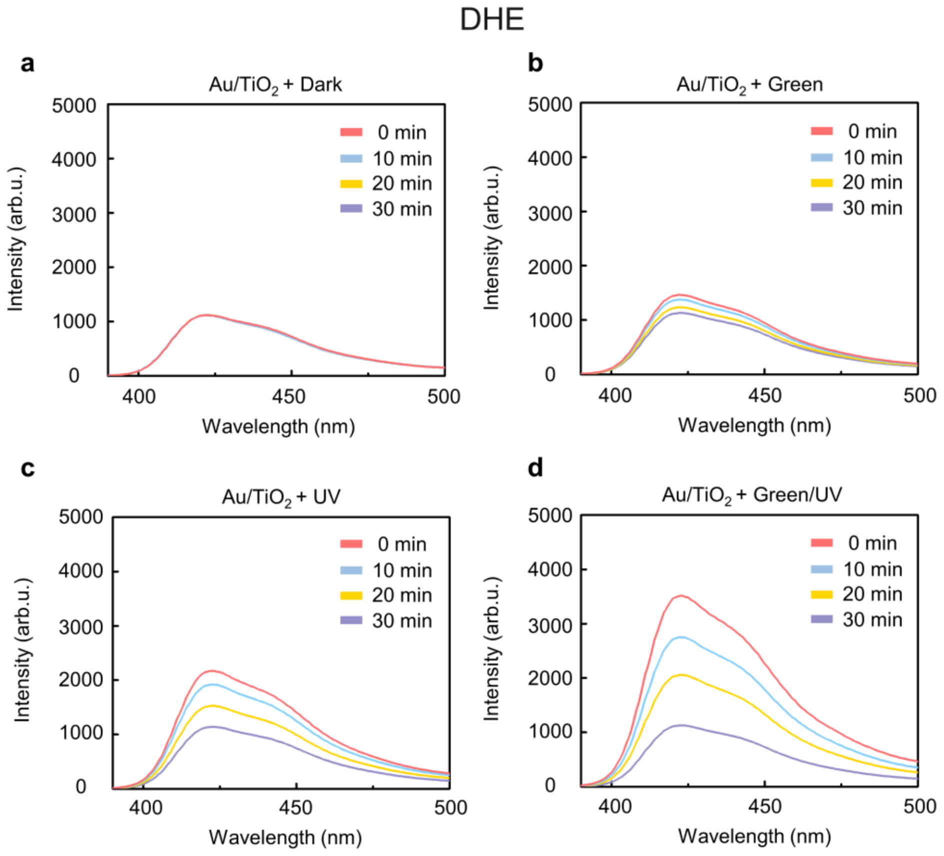

Supplementary Fig. 8. **Fluorescence intensity of DHE.** Time-dependent fluorescence intensity of DHE for detecting the $O_2^{-\bullet}$ generation of the Au/TiO_2 heterostructure irradiated by **a** dark, **b** green, **c** UV, and **d** green/UV. arb.u., arbitrary units. Colors in red, blue, yellow, and purple represent 0 min, 20 min, 40 min, and 60 min irradiation, respectively. Source data are provided as a Source Data file.

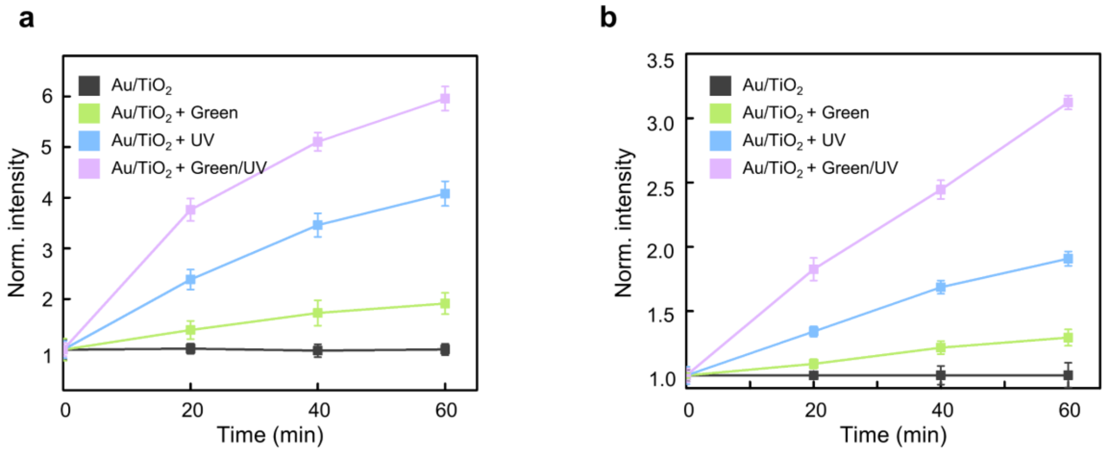

Supplementary Fig. 9. Normalized intensities of 7-OH-coumarin and DHE under green, UV, and green/UV irradiation. Time-dependent fluorescence intensity of **a** 7-OH-coumarin at 455 nm and **b** DHE at 420 nm. Data are expressed as mean ± SD ($n = 3$ independent experiments). Colors in gray, green, blue, and purple represent Au/TiO_2, Au/TiO_2 + green, Au/TiO_2 + UV, and Au/TiO_2 + green/UV irradiation, respectively. Source data are provided as a Source Data file.

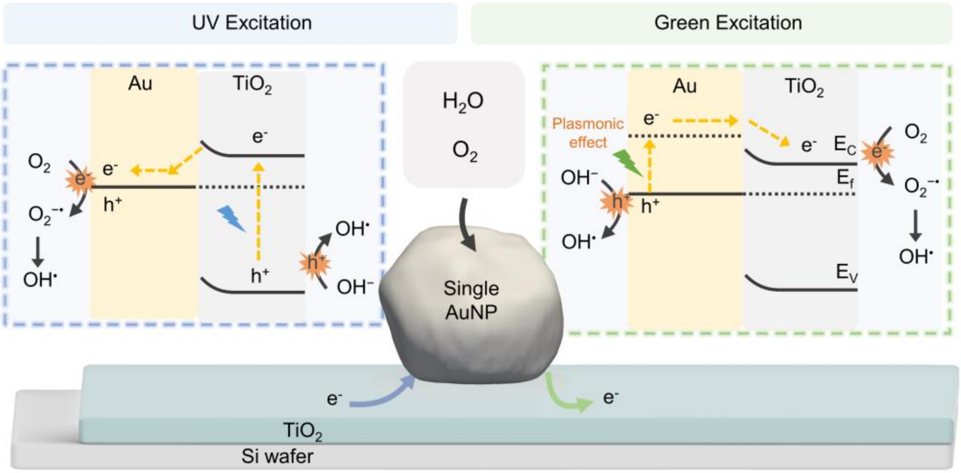

Supplementary Fig. 10. Schematic illustration of the proposed photocatalytic mechanism of Au/TiO_2 heterostructure under green and UV light irradiation.

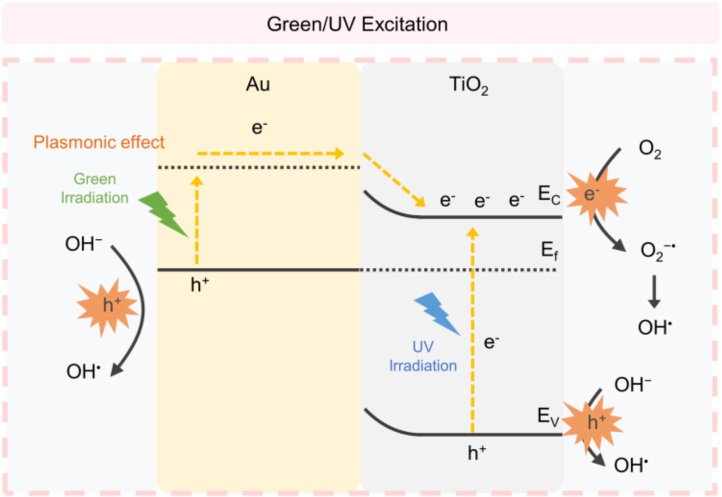

Supplementary Fig. 11. Schematic illustration of the proposed photocatalytic mechanism of Au/TiO_2 heterostructure under green/UV irradiation.

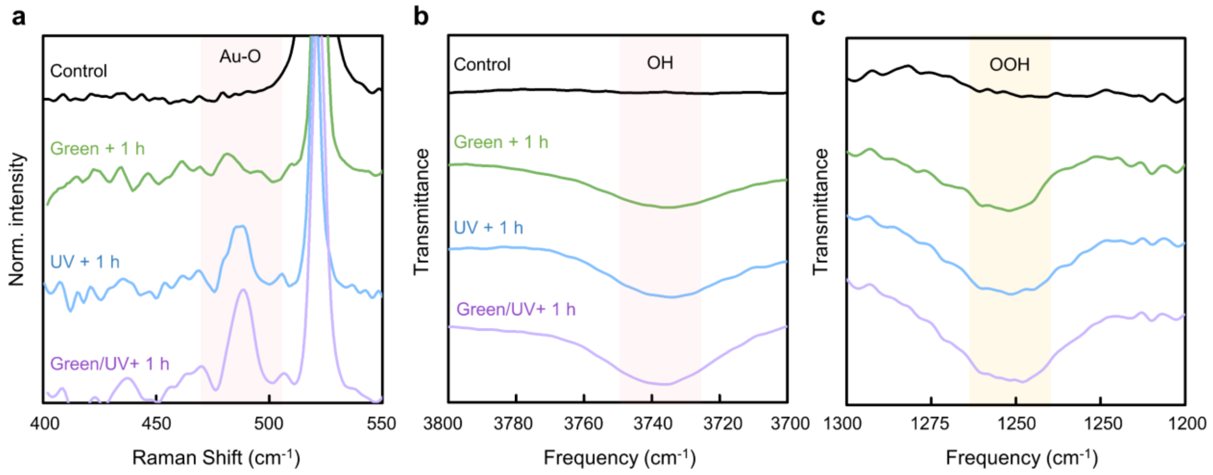

Supplementary Fig. 12. Characterization of ROS adsorption on the AuNP surface. a Raman spectra of Au-O for the Au/TiO_2 after green, UV, and green/UV irradiation, respectively. FT-IR spectra of **b** OH and **c** OOH bond for the Au/TiO_2 under green, UV, and green/UV irradiation, respectively. Colors in gray, green, blue, and purple represent control, green, UV, and green/UV irradiation, respectively. Source data are provided as a Source Data file.

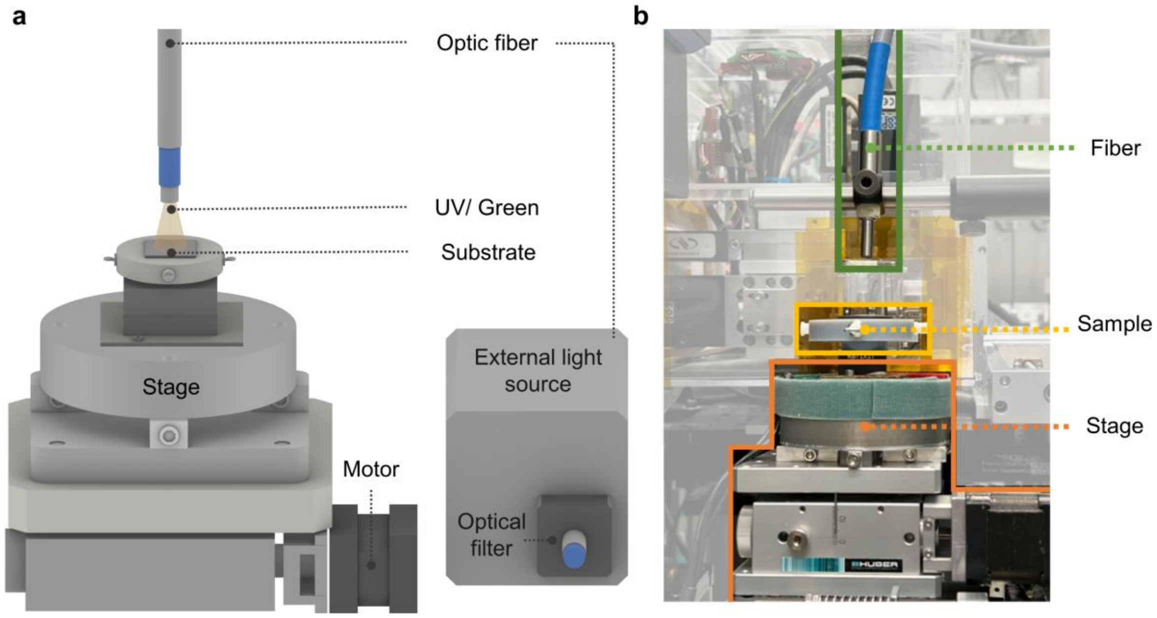

Supplementary Fig. 13. Experimental setup to image strain distribution onto a single AuNP. a Schematic view of the of in-situ photocatalytic BCDI setup. **b** Photograph of the in-situ photocatalytic BCDI setup at the sector 34-ID-C at APS.

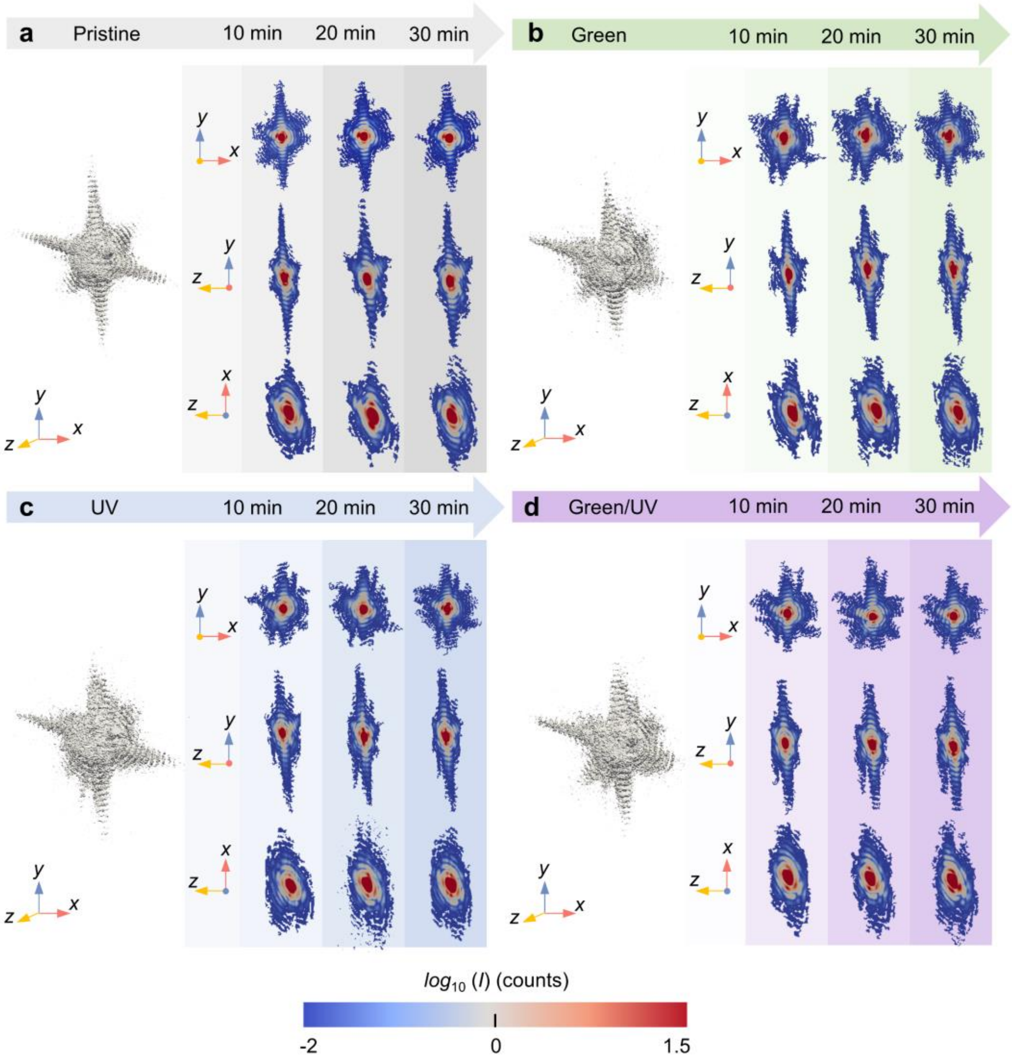

Supplementary Fig. 14. Coherent x-ray diffraction measurements of the AuNP at the (111) Bragg peak as a function of *x*, *y*, and *z* coordinates of the scattering vector Q_{111}. The intensity has been normalized by its maximum value. The illumination conditions are **a** pristine, **b** green, **c** UV, and **d** green/UV. As light irradiation continued, leading to the generation of ROS, slight changes were observed in the center of the Bragg peak, and the fringe pattern developed a pronounced asymmetry, whereas the pristine condition exhibited minimal change over time.

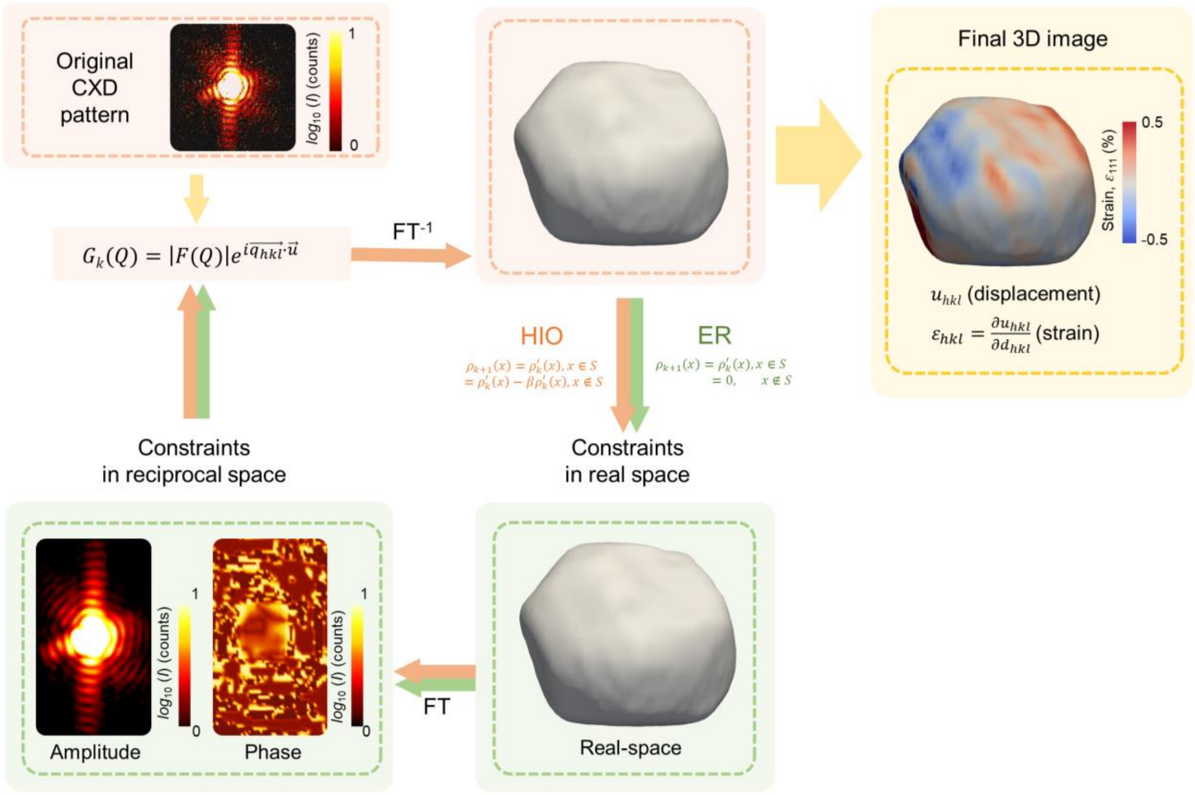

Supplementary Fig. 15. Schematic diagram of phase retrieval algorithm combined with error reduction (ER) and hybrid input-output (HIO) using coherent diffraction pattern.

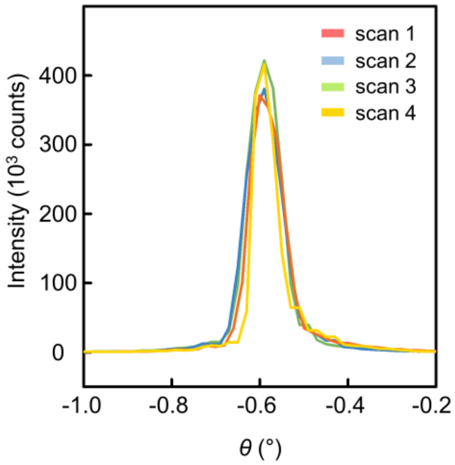

Supplementary Fig. 16. Bragg peak intensity as a function of rocking curve angle for successive scans of the AuNP in air condition. Source data are provided as a Source Data file.

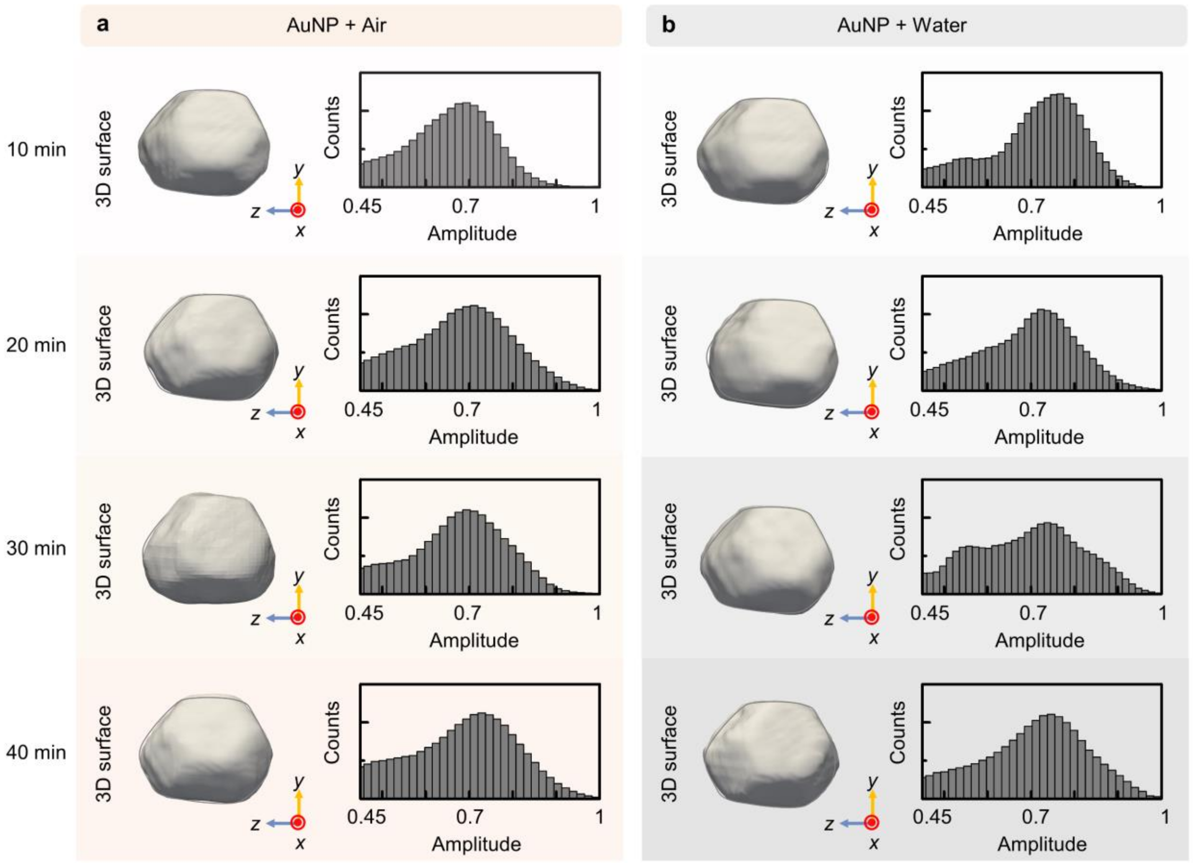

Supplementary Fig. 17. Amplitude variation of the AuNP under air and water conditions. 3D reconstructed images and corresponding amplitude distribution of a single AuNP measured in **a** air and **b** water conditions. The gray boundary illustrates the shape of the AuNP + air at 10 min. Source data are provided as a Source Data file.

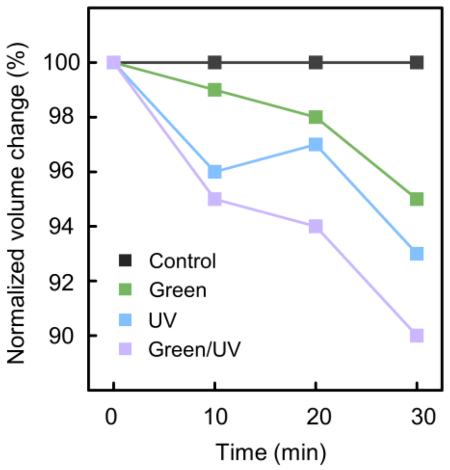

Supplementary Fig. 18. Volume changes of the AuNP as a function of irradiation time under green, UV, and green/UV irradiation. The volume of the AuNP is evaluated from the 3D reconstructed images. Colors in gray, green, blue, and purple represent control, green, UV, and green/UV irradiation, respectively. Source data are provided as a Source Data file.

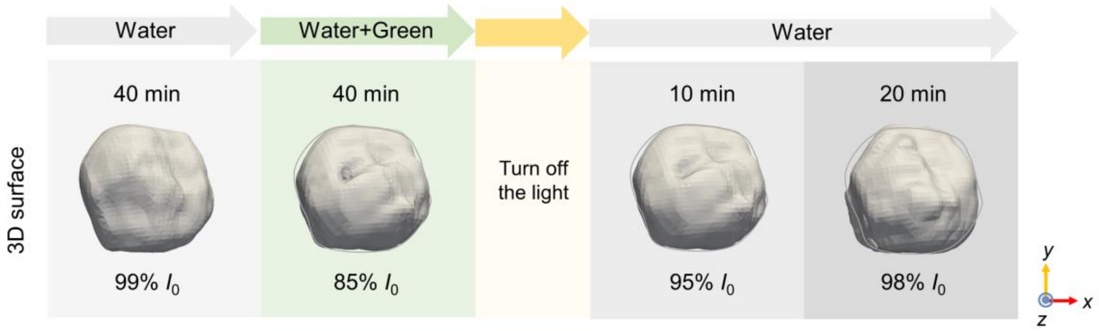

Supplementary Fig. 19. Structural deformation of a single AuNP as a function of light irradiation. As the irradiation time increased, the integrated intensity of Bragg peak decreased to 85%, and voids were observed inside the AuNP. After turning off the light, the intensity recovered up to 98%. 3D volume viewed at a 30% amplitude threshold. The gray boundary illustrates the shape of the AuNP in pristine condition at 40 min.

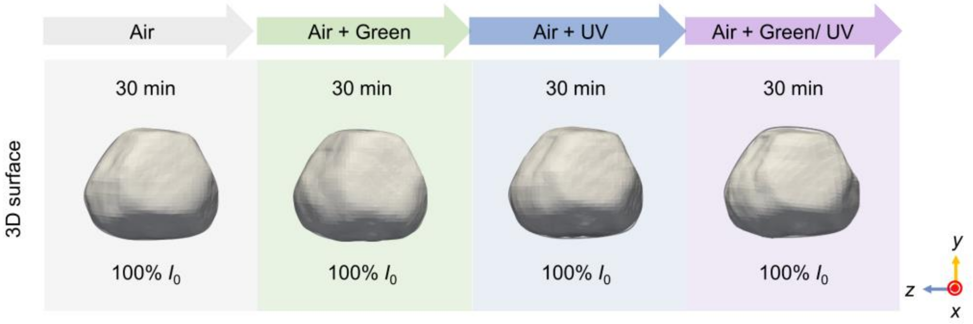

Supplementary Fig. 20. Morphology and strain distribution changes of AuNP in air. 3D volume viewed at a 30% amplitude threshold. The gray boundary illustrates the shape of the AuNP + air in pristine condition at 30 min.

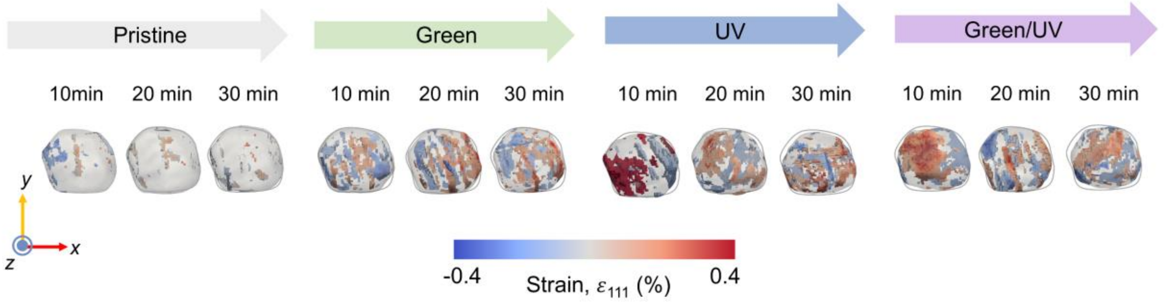

Supplementary Fig. 21. 3D strain images depicting the highly compressive (blue, strain < -0.2% shown in Fig. 3b) and tensile (red, strain > 0.2% shown in Fig. 3b) strain regions during the photocatalytic reaction. The particle shape is shown as a semi-transparent grey isosurface. The gray boundary illustrates the shape of the AuNP in pristine condition at 10 min.

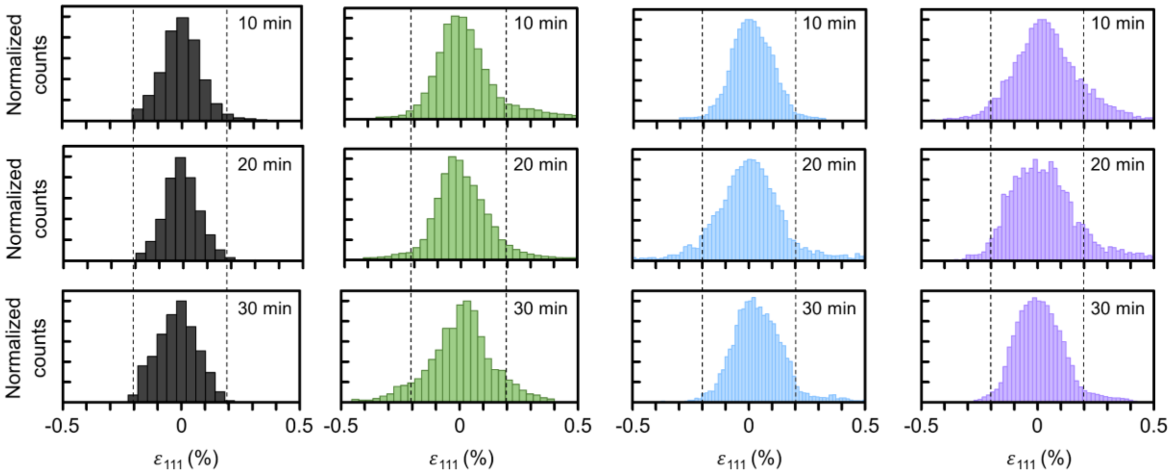

Supplementary Fig. 22. Surface strain distribution of the AuNP from Fig. 2. The lines on the strain plots indicate the threshold values (-0.2% and 0.2%). Colors in gray, green, blue, and purple represent pristine, green, UV, and green/UV irradiation, respectively. Source data are provided as a Source Data file.

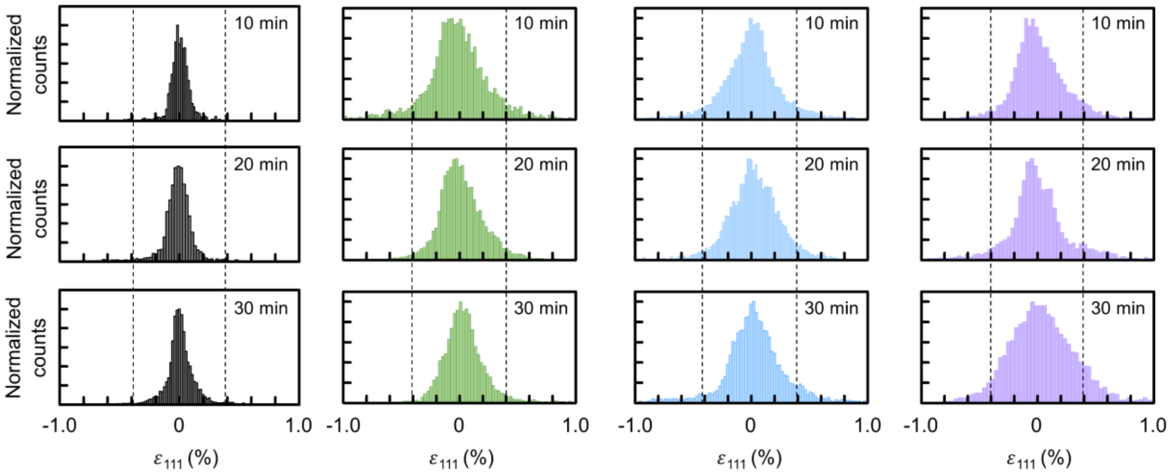

Supplementary Fig. 23. Surface strain distribution of a single ellipsoidal AuNP. The lines on the strain plots indicate the threshold values (-0.4% and 0.4%). Colors in gray, green, blue, and purple represent pristine, green, UV, and green/UV irradiation, respectively. Source data are provided as a Source Data file.

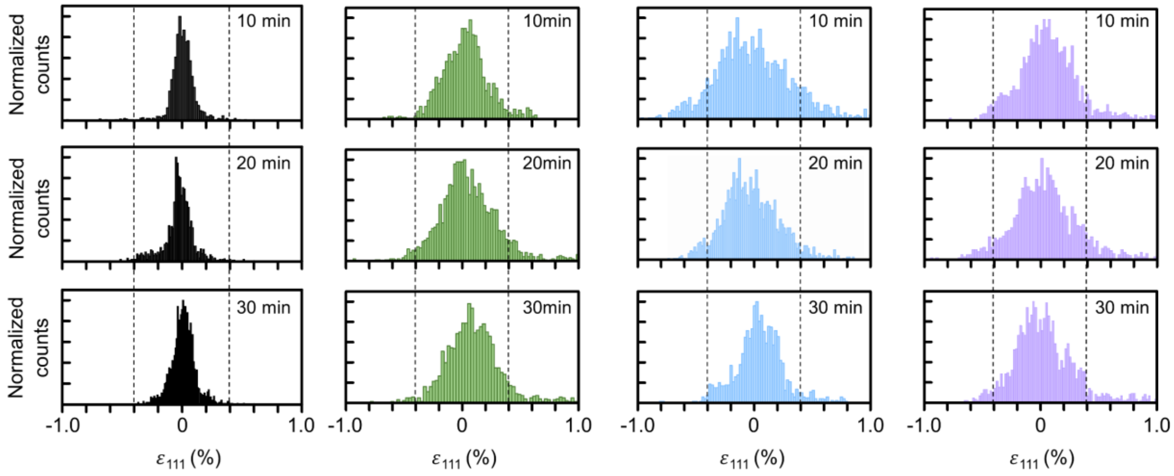

Supplementary Fig. 24. Bulk strain distribution of a single ellipsoidal AuNP. The lines on the strain plots indicate the threshold values (-0.4% and 0.4%). Colors in gray, green, blue, and purple represent pristine, green, UV, and green/UV irradiation, respectively. Source data are provided as a Source Data file.

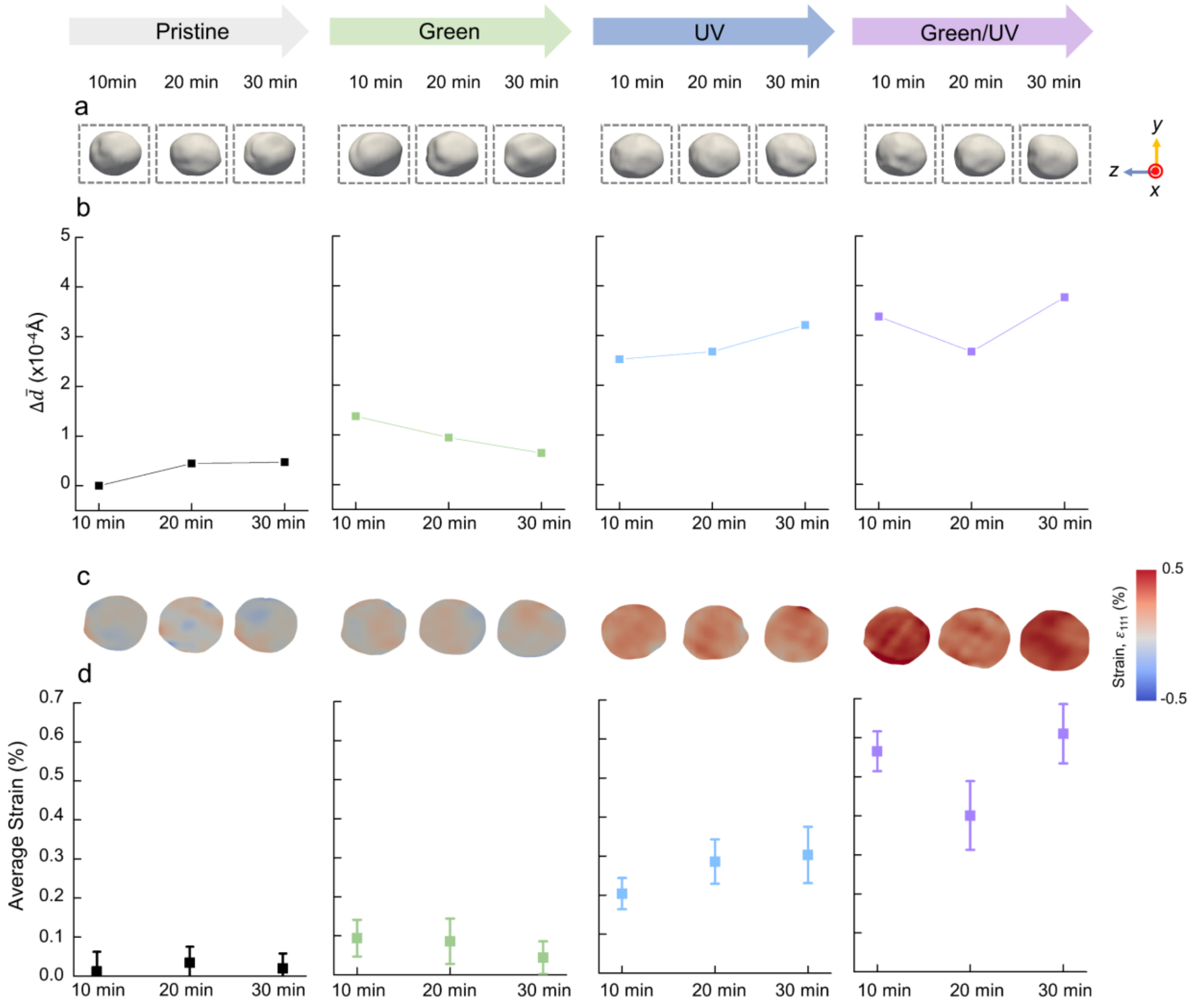

Supplementary Fig. 25. Average strain field of a single AuNP. a 3D reconstructed images at a 30% amplitude threshold with lattice displacement along the [111] direction projected on the isosurfaces. The gray boundary depicts the pristine cross-section in Supplementary Fig. 25a. **b** The change in $\Delta\bar{d}$ is calculated using the formula $\Delta\bar{d} = \bar{d}_{111} - \bar{d}^{*}_{111}$, where the $\bar{d}_{111}$ is the average lattice spacing measured across the AuNP and $\bar{d}^{*}_{111}$ is the average lattice spacing of the first measured pristine AuNP. **c** Cross-sectional views of the internal strain field at the dashed line box in Supplementary Fig. 25a. **d** Average strain field for the internal plane in Supplementary Fig. 25c under green, UV, and green/UV irradiation for 30 min. The gray boundary illustrates the shape of the AuNP in pristine condition at 10 min. The error bars represent the standard deviation of the strain of AuNP. Colors in gray, green, blue, and purple represent pristine, green, UV, and green/UV irradiation, respectively. Source data are provided as a Source Data file.

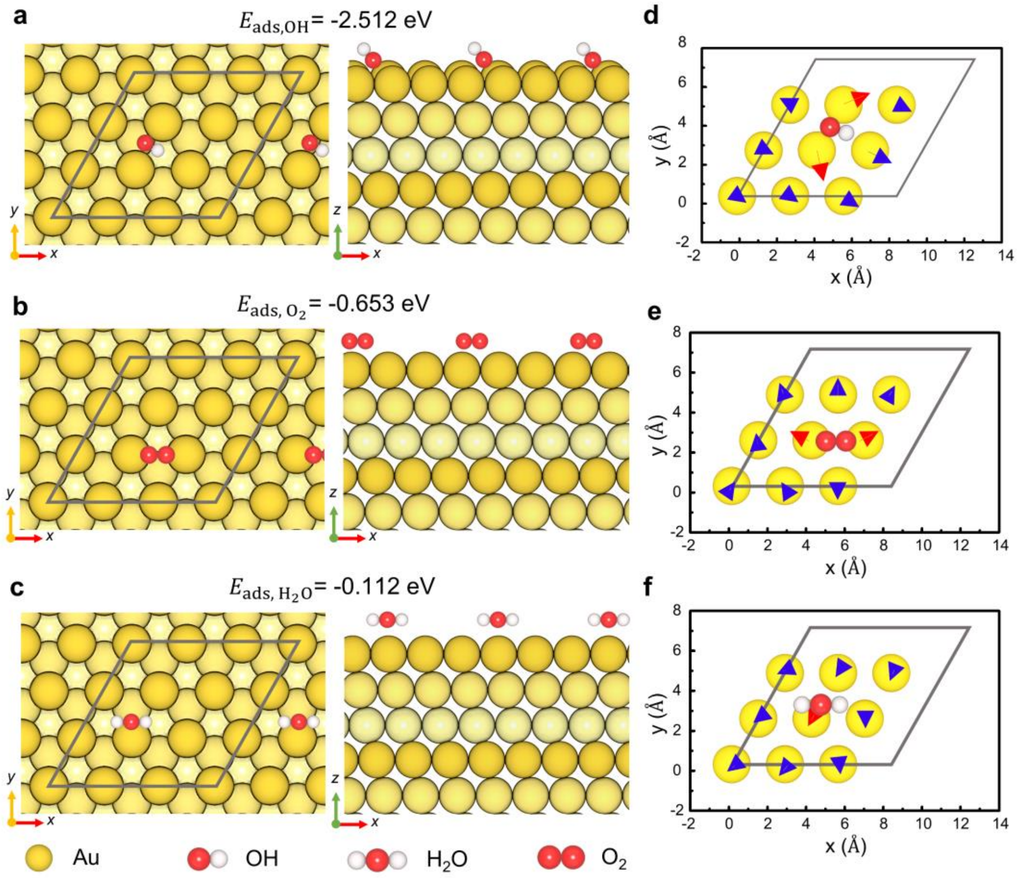

Supplementary Fig. 26. Adsorption configurations of OH, O_2, and H_2O on AuNP and their associated displacement response. a-c Optimized adsorption configurations of OH, O_2, and H_2O on the Au (111) surface: (left) top view and (right) side view of the most stable structure. **d-f** In-plane distortion of the top-layer Au atoms for the adsorption of OH, O_2, and H_2O. The in-plane distortion of the top-layer Au surface is determined from the top 9 Au atoms. The red and blue colors represent the out-of-plane directions along the *z*-axis, with red indicating upward movement in the +*z* direction away from the center of mass, and blue indicating downward movement in the -*z* direction toward the surface. Source data are provided as a Source Data file.

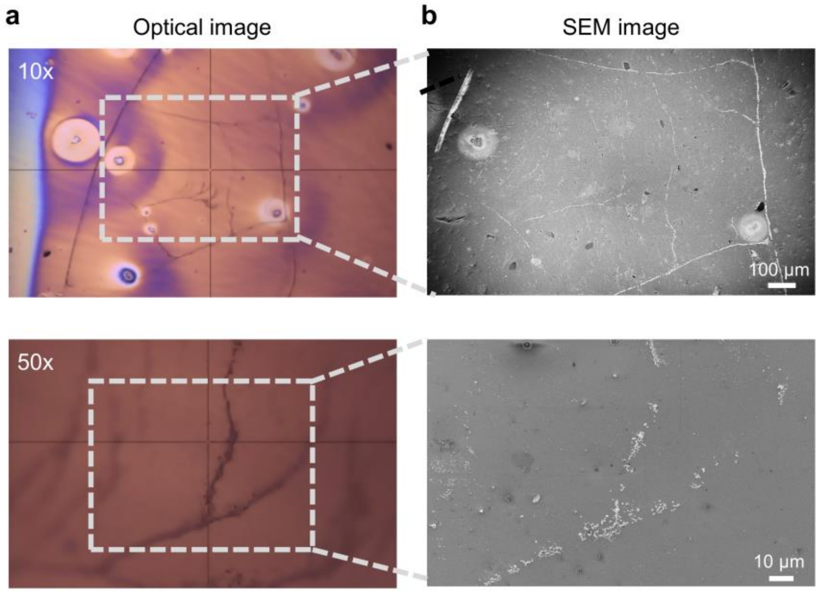

Supplementary Fig. 27. Overview topography image with measured nanoparticles. a Optical images of Au/TiO_2 heterostructures taken from the optical microscope mounted on the beamline. **b** SEM images of the squared region in optical images.

	Water	Green	Water	
	40 min	40 min	10 min	20 min
(a) Average Strain (%)	0.05	0.23	0.12	0.11
(b) $\Delta\bar{d}$ (10^{-3} Å)	0.01	1.92	0.15	0.18

Supplementary Table 1. Structural variation of AuNP under water and green condition. **a** Average strain field of the AuNP in Supplementary Fig 19. **b** Variation in the average lattice spacing under water and green condition. The change in $\Delta\bar{d}$ is calculated using the formula $\Delta\bar{d} = \bar{d}_{111} - \bar{d}^{*}_{111}$, where the $\bar{d}_{111}$ is the average lattice spacing measured across the AuNP and $\bar{d}^{*}_{111}$ is the average lattice spacing of the first measured pristine AuNP.

FWHM (%)	Pristine		Green		UV		Green/UV	
	Surface	Bulk	Surface	Bulk	Surface	Bulk	Surface	Bulk
10 min	0.018	0.015	0.023	0.021	0.021	0.020	0.026	0.022
20 min	0.017	0.016	0.022	0.022	0.025	0.022	0.028	0.023
30 min	0.019	0.019	0.023	0.020	0.024	0.021	0.025	0.021
Average	0.018	0.017	0.022	0.021	0.023	0.021	0.026	0.022

Supplementary Table 2. Full width at half maximum value of surface versus bulk strain distributions as a function of the irradiated wavelength.

Sample		Spatial resolution			
		Voxel size (nm^3)	*x* direction (nm)	*y* direction (nm)	*z* direction (nm)
AuNP (shown in Fig 2)	Pristine (10 min)	4.40 × 4.40 × 4.40	11.0	8.9	15.6
	Pristine (20 min)	4.40 × 4.40 × 4.40	9.2	8.6	14.3
	Pristine (30 min)	4.40 × 4.40 × 4.40	11.5	9.4	17.4
	Pristine (40 min)	4.40 × 4.40 × 4.40	10.1	8.0	16.4
	Green (10 min)	4.40 × 4.40 × 4.40	9.5	9.7	13.0
	Green (20 min)	4.40 × 4.40 × 4.40	13.3	12.2	16.0
	Green (30 min)	4.40 × 4.40 × 4.40	12.2	10.0	17.3
	UV (10 min)	4.40 × 4.40 × 4.40	9.9	8.0	16.6
	UV (20 min)	4.40 × 4.40 × 4.40	11.3	8.2	17.8
	UV (30 min)	4.40 × 4.40 × 4.40	11.4	9.0	15.6
	Green/UV (10 min)	4.40 × 4.40 × 4.40	9.7	8.1	13.3
	Green/UV (20 min)	4.40 × 4.40 × 4.40	10.5	9.4	10.9
	Green/UV (30 min)	4.40 × 4.40 × 4.40	18.9	13.4	17.4
Ellipsoidal AuNP (shown in Supplementary Figs. 23-25)	Pristine (10 min)	5.18 × 5.18 × 5.18	11.8	10.2	17.2
	Pristine (20 min)	5.18 × 5.18 × 5.18	12.6	10.5	17.1
	Pristine (30 min)	5.18 × 5.18 × 5.18	11.3	9.3	15.5
	Green (10 min)	5.18 × 5.18 × 5.18	12.2	9.9	18.6
	Green (20 min)	5.18 × 5.18 × 5.18	12.8	10.0	19.9
	Green (30 min)	5.18 × 5.18 × 5.18	14.2	11.8	15.1
	UV (10 min)	5.18 × 5.18 × 5.18	11.6	9.5	15.3
	UV (20 min)	5.18 × 5.18 × 5.18	11.6	9.9	17.4
	UV (30 min)	5.18 × 5.18 × 5.18	13.2	11.5	13.4
	Green/UV (10 min)	5.18 × 5.18 × 5.18	13.1	10.8	17.5
	Green/UV (20 min)	5.18 × 5.18 × 5.18	11.4	11.2	14.7
	Green/UV (30 min)	5.18 × 5.18 × 5.18	13.8	10.8	16.2

Supplementary Table 3. Spatial resolution of AuNPs in the *x*, *y*, and *z* directions for each measurement condition.

Supplementary Note 1. X-ray stability of AuNP during BCDI measurements

1) The change in crystallinity of the pristine AuNP: The particle stability was confirmed by repeatedly measuring the rocking curve of the single AuNP in air, indicating the NP was well fixed to the substrate (Supplementary Fig. 16). The stability was also confirmed as both the amplitude histogram and the particle shape remained constant for 40 min in air (Supplementary Fig. 17a). To show the particle stability in the water environment, BCDI data collected at 40 min were added to the pristine results of Fig. 3a (Supplementary Fig. 17b). A slight difference was observed in the amplitude histogram measured at 30 min in the water environment. However, it is important to note that the particle shape and amplitude histogram measured at 40 min returned to their original state. This suggests that the observed change was likely temporary, possibly due to momentary beam fluctuations or a slight misalignment of the AuNP. Therefore, even under long-term X-ray beam irradiation, the particle shape and amplitude histogram in the water environment remained nearly identical to those observed in air, indicating that the NP is stable during BCDI measurements.

2) Water radiolysis and photo-ionization: As shown in Fig. 2l, the slight strain change observed on the Au surface over time under pristine conditions can be attributed to the adsorption of ROS, which is generated through water radiolysis, onto the Au surface. However, the amount of ROS generated near the Au surface during only X-ray irradiation is extremely small, and the majority is scavenged by dissolved oxygen or impurities[1]. As shown in Supplementary Figs. 19 and 20, when irradiated with a green light in the water environment, the integrated intensity of the Bragg peak decreased to 85% of I_0. In contrast, both under green light irradiation in air and in the absence of light in air, the integrated intensity shows no change, remaining at I_0. The stability of the diffraction intensity and volume across each scan suggests that neither water radiolysis nor photo-ionization induces significant changes in the nanoparticle.

Supplementary Reference

1. Le Caër S. Water Radiolysis: Influence of Oxide Surfaces on H_2 Production under Ionizing Radiation. *Water* **3**, 235-253 (2011).
